# A Feasibility Randomised Controlled Trial of the New Orleans Intervention for Infant Mental Health: A Study Protocol

**DOI:** 10.1155/2013/838042

**Published:** 2013-04-23

**Authors:** Rachel Pritchett, Bridie Fitzpatrick, Nicholas Watson, Richard Cotmore, Philip Wilson, Graham Bryce, Julia Donaldson, Kathleen Boyd, Charles Zeanah, John Norrie, Julie Taylor, Julie Larrieu, Martina Messow, Matt Forde, Fiona Turner, Susan Irving, Helen Minnis

**Affiliations:** ^1^Academic Unit of Mental Health & Wellbeing, University of Glasgow, Caledonia House, Royal Hospital for Sick Children, Glasgow G3 8SJ, UK; ^2^College of Medical, Veterinary and Life Sciences, University of Glasgow, General Practice and Primary Care, 1 Horselethill Road, Glasgow G12 9LX, UK; ^3^Strathclyde Centre for Disability Research, Institute for Health and Wellbeing, School of Social and Political Sciences, University of Glasgow, Adam Smith Building, 40 Bute Gardens, Glasgow G12 8RT, UK; ^4^NSPCC, Weston House, 42 Curtain Road, London EC2A 3NH, UK; ^5^Centre for Rural Health, University of Aberdeen, Centre for Health Science, Old Perth Road, Inverness IV2 3JH, UK; ^6^Glasgow Infant and Family Team, NSPCC Scotland, Rowanpark, Ardlaw Street, Glasgow G51 3RR, UK; ^7^Health Economics & Health Technology Assessment, Institute of Health & Wellbeing, University of Glasgow, 1 Lilybank Gardens, Glasgow G12 8RZ, UK; ^8^Tulane University School of Medicine, 1430 Tulane Avenue, No. 8055, New Orleans, LA 70112, USA; ^9^Health Services Research Unit, 3rd Floor Health Sciences Building, University of Aberdeen, Foresterhill, Aberdeen AB25 2ZD, UK; ^10^Child Protection Research Centre, University of Edinburgh, St Leonard's Land, Holyrood Road, Edinburgh EH8 8AQ, UK; ^11^Robertson Centre for Biostatistics, University of Glasgow, Level 11, Boyd Orr Building, University Avenue, Glasgow G12 8QQ, UK; ^12^NSPCC Scotland, 2nd Floor, Tara House, 46 Bath Street, Glasgow G2 1HG, UK; ^13^Academic Unit of Mental Health and Wellbeing, Institute of Health and Wellbeing, University of Glasgow, Gartnavel Royal Hospital, 1055 Great Western Road, Glasgow G12 0XH, UK

## Abstract

Child maltreatment is associated with life-long social, physical, and mental health problems. Intervening early to provide maltreated children with safe, nurturing care can improve outcomes. The need for prompt decisions about permanent placement (i.e., regarding adoption or return home) is internationally recognised. However, a recent Glasgow audit showed that many maltreated children “revolve” between birth families and foster carers. This paper describes the protocol of the first exploratory randomised controlled trial of a mental health intervention aimed at improving placement permanency decisions for maltreated children. This trial compares an infant's mental health intervention with the new enhanced service as usual for maltreated children entering care in Glasgow. As both are new services, the trial is being conducted from a position of equipoise. The outcome assessment covers various fields of a child's neurodevelopment to identify problems in any ESSENCE domain. The feasibility, reliability, and developmental appropriateness of all outcome measures are examined. Additionally, the potential for linkage with routinely collected data on health and social care and, in the future, education is explored. The results will inform a definitive randomised controlled trial that could potentially lead to long lasting benefits for the Scottish population and which may be applicable to other areas of the world. This trial is registered with ClinicalTrials.gov (NC01485510).

## 1. Background

Child maltreatment is known to be associated with significant problems in later life affecting both physical [1] and mental health [2–4]. Intervening early can improve outcomes when children's social and emotional development is at risk [5], and recovery from the effects of maltreatment is possible if children are provided with safe and nurturing care early, ideally in the first year of life [6–8]. Failure to do so puts children at risk of disrupted attachments and poor emotional well-being [9]. There is a growing international research and policy consensus on the need for prompt decisions about permanent placement (i.e., regarding adoption or return home) so that children can experience secure care as early as possible [9–14]. However, a recent audit of services in Glasgow revealed that children frequently “revolve” between maltreating birth parents and various temporary foster placements [10]. Moreover, there are no infant mental health services focusing on maltreated infants in Scotland.

There have been attempts to develop interventions to improve the mental health of maltreated infants [6, 7], but only one evaluated programme was identified, the Tulane Infant Team in New Orleans, Louisiana [8]. This is still in operation and aims to improve the permanency decision-making process using a comprehensive mental health intervention. Permanency decisions involve placing a child in the care of one family until the child reaches the age of independence. The Tulane Infant Team offers a tailored intervention to every family with a child coming into care. It assesses the quality of child's relationships and the degree of change over the course of the intervention. It makes considered recommendations to inform the legal system about the best placement outcome for each child. The aim of the Tulane Infant Team is to rehabilitate children back to their birth parents, and when this cannot be achieved safely or quickly enough, to free the children for adoption. An evaluation based on analysis of routine data was conducted four years prior to and four years after the introduction of this intervention to New Orleans [8]. This suggested that more children were adopted following its introduction; however, for those returned to birth families, there was a significant reduction in repeated maltreatment both for that child and subsequent siblings. The limitation of the study was a consecutive cohort design, and the lack of randomisation means that factors other than the intervention may be contributing to the positive effects. 

Deciding which are the most appropriate outcome measures for this population is challenging. Gillberg [11] described the high levels of coexistence between symptoms of different disorders in early childhood, which he defined as ESSENCE (Early Symptomatic Syndromes Eliciting Neurodevelopmental Clinical Examinations). This demonstrates the need for a diverse and thorough assessment across the various fields described by Gillberg [11], including general development, language, social interrelatedness, mood, and behaviour. 

The overall aim of this exploratory randomised controlled trial (RCT) is to evaluate the feasibility and to inform the design of a definitive RCT evaluating a Scottish adaptation of the New Orleans intervention for maltreated children. The specific research questions are as follows:

1. what are the size and nature of any effects of the Glasgow version of the New Orleans model, the Glasgow Infant and Family Team (GIFT), on the mental health of maltreated preschool children?
2. is a definitive multicentre RCT of GIFT feasible, acceptable, and necessary?
3. what would be the required size of a definitive RCT of GIFT?
4. what would be the optimal outcome measures for a definitive RCT of GIFT?
5. what are the beliefs, attitudes, and experiences of those managing and delivering GIFT and an enhanced usual service, the Family Assessment and Contact Service (FACS)?
6. is GIFT likely to be cost-effective in Glasgow and, if so, what design parameters are required for a definitive RCT?

## 2. Methods/Design

### 2.1. Study Design and Setting

This study is an RCT comparing two arms: GIFT (the intervention arm) and FACS (enhanced version of services as usual arm). Outcome measures from all participants are being collected one month after a child comes into care and then again one year later.

The study is set in the city of Glasgow, Scotland's largest and most ethnically diverse city with an estimated population of 588,470 of which almost 6% represent an ethnic minority (http://www.glasgow.gov.uk/index.aspx?articleid=3969). Children represent 16% of the Glasgow population; over one-third live in areas in the most deprived decile within Scotland, whilst only 3% live in areas in the least deprived decile within Scotland.

### 2.2. Participants

All parents (or recognised parental guardians) with a child aged between 6 and 60 months who come into a period of care due to child protection concerns are invited to take part in the study. Children are excluded from the study if

1. they have a profound learning disability (as assessment outcome measures would not be appropriate),
2. and/or their primary caregiver is unavailable to take part in the intervention (such as long-term imprisonment, death, or being uncontactable by services or research team for 3 months or more).

### 2.3. Recruitment and Randomisation

Recruitment is taking place over 17 months from December 2011 to April 2013. An estimated 153 eligible children are expected to enter care due to maltreatment during this period, that is, 9 children per month. Consent from parents and foster carers to be approached by the research team to discuss the study is obtained by the social worker who gives the potential participants an information leaflet and a digital video disc explaining the study, its intent, and what participation would entail. Thereafter, informed consent from those agreeing to be contacted is obtained by the study's recruitment officer. 

**Figure 1:**
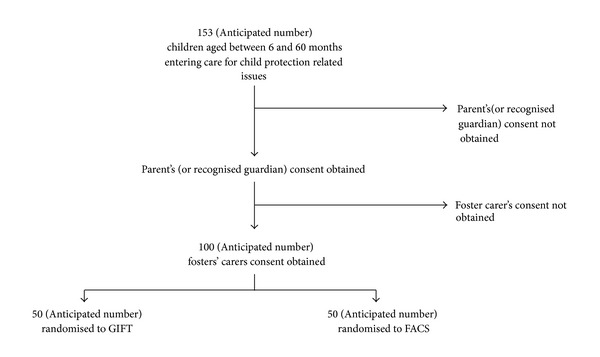
Anticipated number of eligible children recruited and randomised in the trial between December 2011 and April 2013.

An anticipated consent rate of 65% will include approximately 100 families in the study, with 50 children in each trial arm (Figure 1). The families are randomly allocated to GIFT or FACS by the Robertson Centre for Biostatistics. Children from the same birth family, regardless of placement, are assigned the same study arm to reduce contamination (as birth parents are the primary target of the intervention). Randomisation is also stratified by child's age (<2 and ≥2 years). The trial arm allocation is concealed from the researchers who carry out the baseline and follow-up assessments, and the first research assessment is carried out prior to randomisation.

### 2.4. Care as Usual: Family Assessment and Contact Service (FACS)

FACS comprises a team of social workers, which undertakes an assessment of the child and the family in order to make a decision about the child's future care. It examines family functioning and makes recommendations regarding placement outcomes for children. It is able to refer family members onto additional services (e.g., drug rehabilitation). Although FACS is an established service in Glasgow, it was previously a specialised team assessing only small numbers of children. As the delivery of early assessment services in Scotland was known to be highly heterogeneous, FACS will offer a new level of consistency and therefore is considered to be “enhanced services as usual”. Any child whose parent or foster carer does not consent to participate in the research study will therefore receive the service from FACS.

### 2.5. The Trial Intervention: Glasgow Infant and Family Team (GIFT)

GIFT is a structured intervention with the primary goal of rehabilitating the child back with their primary caregiver, when it is safe to do so. The team is multidisciplinary incorporating social workers, psychologists, a psychotherapist, and a psychiatrist. Like FACS, GIFT makes an assessment of the children in the context of their relationships with their caregivers. Whilst both teams assess relationships with the birth parents, GIFT also always assesses the relationships with foster carers. GIFT arranges referrals onto other services as described in FACS. GIFT also offers an intensive relationship focussed intervention to every birth family, which is anticipated to take between 6 and 9 months. This intervention is aimed at improving the relationship between the child and his/her birth family and according to the outcome, GIFT recommends whether the children should return home or be adopted. The intention is that all foster carers who care for children coming to the GIFT intervention should be jointly registered as potential adopters so that, if rehabilitation home is not feasible, the child does not have to experience another change of placement before achieving permanency. However, it is likely that this will take time to achieve and that not all carers will be dually registered within the recruitment period.

### 2.6. Outcome Measures

Baseline assessment based on the outcome measures is administered at a minimum of one month after the child is received into care. One month is allowed to let the carer get to know the child as well as to allow for the child to settle into the carer's home. Follow up assessment of the outcome measures is then repeated one year later. At baseline, the assessment is completed for all children with their foster carers. At follow up, the assessment is completed with the child's primary caregiver at that time who may be the birth parent, adoptive parent, or the foster carer—who may be the same or different from the foster carer at baseline. 

### 2.7. Primary Outcome Measure

Infant mental health is measured using the Infant-Toddler Social and Emotional Assessment (ITSEA) [12, 13]. This 166-item questionnaire is well validated and is completed by the parent or carer [14]. It covers a wide range of social and emotional behaviours in infants, across four domains: externalising, internalising, dysregulation, and competence. It has been used successfully in previous interventions research with maltreated children showing medium to large effect sizes and good longitudinal stability [15]. 

### 2.8. Secondary Outcome Measures

A cognitive assessment of the child is undertaken. Children under 2.5 years are assessed with the Bayley Scales of Infant Development [16], while children 2.5 years and over are assessed using the Wechsler Preschool and Primary Scale of Intelligence (WPPSI IV) [17]. The parent or carer also completes the Parent Evaluation of Development Status (PEDS) [18] which assesses cognitive milestones including language, the Disturbances of Attachment Interview (DAI) [19] which identifies symptoms of attachment disorders, the Parent-Infant Relationship Global Assessment of Functioning (PIR-GAS) [20] which assesses global relationship functioning following observation of both play and meal time activities, and the Paediatric Quality of Life Inventory (PedsQL) [21] which assesses health-related quality of life. The Development and Well-being Assessment (DAWBA) [22] is completed by carers with a child aged two and above and is used to generate International Classification of Diseases (ICD) and Diagnostic and Statistical Manual of Mental Disorders (DSM) codes. The Waiting Room Observation (WRO) [23], a structured observation for symptoms of attachment disorders, is also completed by the researcher when the child and carer first arrive at the clinic. In addition, the Strange Situation Procedure (SSP) [24], the gold standard measure of infant/toddler attachment patterns, is completed at the follow-up time only. 

In addition, the “This Is My Baby” (TIMB) [25] interview, which assesses the degree of commitment to the child by the foster carer is included as it may be investigated as a potential moderator between maltreatment and outcome.

In this exploratory trial, the feasibility, reliability, and developmental appropriateness of each measure will be examined in order to select the best measures for a definitive trial. In addition, the potential for linkage of data with routinely collected data on health and social care and, in the future, education and legal services will be explored. 

### 2.9. Data and Statistical Analysis

The study will be analysed using the intention-to-treat analysis. To make preliminary assessment of the efficacy of GIFT, changes in scores on the ITSEA competence scale will be compared between the GIFT and FACS groups, adjusting for important baseline variables. To assess feasibility, the percentage consenting and the retention rate will be estimated: whether these are related to social circumstances or the type of intervention will be investigated using routine data where possible. 

### 2.10. Treatment Fidelity

A fidelity monitoring model will be tailored to the specific needs of this complex intervention. This aims to capture five key components of fidelity [26] encompassing adherence to the prescribed intervention (staff supervision, training, and participant attendance), exposure (volume of trial intervention received per family), quality of delivery (monitoring assessment and treatment reports, focus group data), responsiveness of families (attendance and case studies), and program differentiation (identifying distinctive features and challenges). The purpose of the model is to ensure that the key components of the intervention are maintained throughout the study, to identify challenges and areas of improvement, and to generate data with which to compare evaluation results with the performance of the intervention.

### 2.11. Health Economics

An economic model will be built and populated with data from the trial to explore the potential cost-effectiveness of GIFT in comparison to FACS, using the ITSEA measure of child's mental health. Child's quality of life will also be measured within trial using the PedsQL for infants and toddlers. Measurement of quality of life is an important input for the economic component of this study and will enable assessment of any short-term change in quality of life for children between baseline and 1 year, and also between the trial arms. The model will be analysed probabilistically in order to characterize uncertainty in the model parameters and estimate confidence limits around the cost and effectiveness outcomes. The economic model will be used to help design the definitive trial proposal.

### 2.12. Qualitative Process Evaluation

Qualitative mapping and modelling work will accompany the exploratory trial in order to track the ways in which FACS and GIFT evolve and impact as services, capturing and exploring issues as they arise throughout the trial and feeding into service development. Qualitative work in the first part of the trial focuses on the implementation and delivery of services from the perspectives of social workers, foster carers, and the GIFT and FACS teams. The main data collection method for this purpose is focus group discussions, which will be repeated throughout the trial in order to track changes and developments over time. The trial consent process is also a focus in this first phase with data being collected from birth parents and foster carers who consent to the study, as well as the professionals responsible for the consent procedure. The second phase of the study, although still tracking the development of issues already gleaned in the first phase, will adopt case study methodology to focus more specifically on the impact of GIFT and FACS on a selection of children and families involved in the trial. This narrower focus will allow an in-depth investigation into the process of experience from the perspectives of the birth family, foster carers, social workers, and health professionals surrounding specific children enrolled in the trial. Case studies will be selected on the basis of a criterion matrix to allow exploration of the experience of receiving both services and different outcomes regarding permanency decisions. Key to this stage of the research is also the gathering of qualitative data from the Children's Hearing System (a panel of specifically trained lay people who, in Scotland, are involved in most child's welfare decisions) in order to explore perspectives about the reports from the service and their impact on decision making. 

The study was approved by the West of Scotland NHS Research Ethics Committee 5 and NHS Greater Glasgow and Clyde (Research and Development Committee). In addition, the research team attended Good Clinical Practice Training and also a study-specific session on obtaining informed consent from very vulnerable families. The protocol was registered before recruitment began on http://www.clinicaltrials.gov/.

## 3. Discussion

Both FACS and GIFT are new services and, during the mapping and modelling phase of this study, it was clear that opinion was divided as to which was likely to provide the best service for maltreated children. We are therefore in a position of equipoise.

The results from the study will provide us with the necessary findings in order to conduct a definitive RCT evaluating the New Orleans intervention for maltreated children. We aim to identify the feasibility of recruiting birth and foster families and the retention of these families to both the research and the interventions. We will assess not only the appropriateness of each measure but the assessments ability to capture problems in any ESSENCE domain. We will also explore the outcomes of the interventions. We will use fidelity monitoring to ascertain and optimise adherence to the GIFT model and to document the delivery of the control intervention. Health economic techniques will be used to assess the implications of such a model in terms of both the costs and outcomes, the results of which will feed into the development and design of a definitive RCT. In addition, we will explore qualitatively the perspectives of those implementing, delivering, and receiving the interventions as part of investigating the feasibility of implementing the model of intervention. In time, we will also examine the impact of the trial on the wider systems through routine data follow up. 

Both GIFT and FACS aim to identify care arrangements which will ensure that the future care of any child who has experienced maltreatment is safe and nurturing. This could potentially lead to a long lasting benefit for the Scottish population as a whole, as well as a reduction in costs to society. Should the GIFT intervention be beneficial to infant's mental health and cost-effective in comparison to FACS, it would be important to consider whether a GIFT intervention could be of benefit in other areas of the UK to improve the life chances of maltreated children and address key policy goals such as improvement of school readiness and community safety. 

### 3.1. Limitations

The GIFT team only has the capacity for a caseload of 50–60 children in one year. This limited capacity means that children removed from parents due to maltreatment but then placed in kinship care, being looked after by family members, are not included. This accounts for a large number of children who are removed from birth parents due to maltreatment. Children in kinship care may be included in future trials.

An additional limitation is that some children will change placement between baseline and follow up, meaning that there will be different respondents. If one intervention proves better at achieving permanent placements than the other, then the number of placement moves is likely to vary between the arms of the trial, thereby introducing bias. This creates a challenge in interpreting results, but the child's primary caregiver at the time is likely to be the best person to report on the child's health and development. 

Children are allocated randomly into GIFT or FACS, and while birth siblings will all be allocated to the same intervention, this will not be possible for nonsiblings placed in the same foster care home. If both birth families and foster families were allocated to the same intervention then this would lead to significant clustering effects; that is, potentially large groups of children (e.g., a large birth sibship spread across several foster homes and all the associated foster sibships) could require randomisation together creating imbalances. Consequently, some foster carers may have children in their care going through both the GIFT and FACS assessments which has the potential to introduce contamination.
